# A decade of minimal access pediatric surgery in India

**DOI:** 10.4103/0971-9261.44760

**Published:** 2008

**Authors:** Anirudh Shah

**Affiliations:** President, Indian Association of Pediatric Surgeons, Amardeep Multispeciality Children Hospital, 65, Pritamnagar Society, Ellisbridge, Ahmedabad - 380006, India. E-mail: dranirudhshah@gmail.com

Pediatric minimal access surgery (MAS) started in India a decade ago and has a great impact on the way we manage pediatric surgical problems today. The imaginations and creativity of pediatric surgeons across the world have lead to a broad array of surgeries that can now be performed without large incisions. New techniques, procedures, and innovations are always ongoing in this fast-developing field. Indications for MAS have remained the same as they were for open surgery. It has a steep learning curve which is not without complications. However, it has the advantages of a shorter hospital stay, less analgesic requirement after surgery, more rapid return to school, and cosmetic satisfaction.

The use of MAS as a diagnostic tool in pediatrics dates back to the 1970’s.[1] With the advances in imaging, the interest in MAS faded. The initial resistance for MAS in children was due to the misconception that smaller patients have smaller incisions, their analgesic requirement is less, and their recovery is speedy as compared to adults. The first laparoscopic cholecystectomy in an adult by Philip Mouret in 1987 rekindled interest in pediatric MAS.[2] Pioneers like Gans, Rodgers, Georgeson, and Lobe showed that MAS was certainly applicable to the pediatric patient.

In India, pediatric MAS started in a major way in 1998 at multiple centers with a series of laparoscopy workshops across the country by the Indian Association of Pediatric Surgeons (IAPS). Prior to this, only sporadic attempts at diagnostic laparoscopy in children were made. Today, a huge amount of pediatric MAS is done, though at limited centers across our country. New centers are rapidly coming up with every passing year. Pediatric endoscopic surgeons of India (PESI) is a subchapter of IAPS formed in the year 2000. Today, MAS has revolutionized the way pediatric surgical problems are managed. MAS has been classified as laparoscopy, thoracoscopy, mediastinoscopy, retroperitoneoscopy, and ventriculoscopy according to the body compartment in which it has been used.

After a decade of work in MAS and a personal experience of almost 3000 cases, time has come to pause, analyze, and group the MAS procedures into the following:

1. MAS as gold standard: MAS has been undoubtedly established as gold standard for evaluation and treatment of impalpable undescended testis, appendicitis, gall stones, early empyema, recurrent abdominal pain, and intersex problems.
2. MAS as controversy:
  - Inguinal hernia: Use of laparoscopy in inguinal hernia is very controversial & is, at present, only an alternative procedure to the time-tested open extraperitoneal repair. Detecting the open contralateral ring remains of doubtful value as clinical manifestations of contralateral hernia are being seen in only about 10–15% in children. High recurrence rate with laparoscopic repair is still unsolved.
  - Pyloromyotomy: laparoscopy has failed to show great advantages over standard open pyloromyotomy which also allows easy repair of the inadvertently opened pyloric mucosa easy.
3. MAS as a boon:
  - Single stage endorectal pull through in Hirschsprung's disease has been widely accepted. Avoiding colostomy in these procedures has saved many children from colostomy related complications.
  - Laparoscopy assisted anorectal pull through (LAARP) is also making an impact and has shown good early results by avoiding division of the striated muscle complex as in PSARP.
4. Difficult MAS: MAS is difficult in pathologies like choledochal cysts, hydronephrosis, large spleens and malignant masses. A vast experience is needed before venturing into these technically difficult procedures. It may be safe to avoid MAS in these pathologies.

There is no doubt that pediatric MAS will continue to expand exponentially, in its indications and applicants. Hospital designs and theatre layouts will change to accommodate this newer trend in surgery. MAS has opened a new, interesting, and intriguing chapter in pediatric surgery. Proper training and dedicated surgical thinking can make MAS safe. The problem is inadequate training centers and trained surgeons. The basic skill of craft has to be acquired from apprenticeship of a master by listening, watching, assisting, being assisted, and then doing independently. Individual operative movements are to be learnt first, before they are amalgamated into a complex action. An increasing number of such fragmented skills are to be perfected outside the operation theatres in an animal lab or in front of an endotrainer before venturing to carry out the procedure on children.

Computers are now routinely used in MAS to record patient information, diagnosis, and treatment. Printers produce copies for inclusion in the patient's notes. Computers allow easy access to information and also provide statistics. This is particularly useful when the need arises to produce evidence or substantiate change. Equipment manufactures are continually improving the design and picture quality of the endoscopes. This not only provides clarity of vision but also allows the assistant, the patient, and his/her relatives to see clearly what is happening. Images can be recorded on tape for later examination or for teaching purposes.

The next 10 years will see electronic developments, for example, the merging of ultrasound and endoscopy. The range of equipments on offer will expand and improve, allowing more freedom of choice and more competitive prices. Accessories are continually being modified to meet the requirements of new MAS procedures. Robotic surgery is a new technology which may expand the variety of operations a surgeon can perform with minimal invasive techniques. Robotic surgery improves MAS through a more natural interface, tremor filtration, motion scaling, and additional degrees-of-freedom of the instruments. The high equipment and procedural cost, however, is restrictive for its use in India at present.

In the future, computer simulation of virtual reality promises to be an excellent method of training in endoscopic procedures. It will provide an alternative method of teaching, requiring no patient involvement. Visual links provided by telecommunication lines could also provide distance learning, allow diagnosis, or second opinion. Microchips or smart cards may be used to hold case notes and be worn by the patient, allowing instant retrieval of medical history, endoscopic findings, etc., when required, in emergency situation or on a routine visit. Despite the many exciting new challenges technology may provide for us, we need to embrace such advances while continuing to provide a high standard of holistic care, tailored to meet individual patients' needs.

With the huge number of patients that we cater to in India, we should also be looking to organize multicenter randomized clinical trails to give a scientific directive on various controversial issues and also validate or challenge the presumed benefits of pediatric MAS in the near future.
